# Liposome-siRNA-Peptide Complexes Cross the Blood-Brain Barrier and Significantly Decrease PrP^C^ on Neuronal Cells and PrP^RES^ in Infected Cell Cultures

**DOI:** 10.1371/journal.pone.0011085

**Published:** 2010-06-14

**Authors:** Bruce Pulford, Natalia Reim, Aimee Bell, Jessica Veatch, Genevieve Forster, Heather Bender, Crystal Meyerett, Scott Hafeman, Brady Michel, Theodore Johnson, A. Christy Wyckoff, Gino Miele, Christian Julius, Jan Kranich, Alan Schenkel, Steven Dow, Mark D. Zabel

**Affiliations:** 1 Department of Microbiology, Immunology and Pathology, College of Veterinary Medicine and Biomedical Sciences, Colorado State University, Fort Collins, Colorado, United States of America; 2 Pfizer Global Research & Development, Translational Medicine Research Collaboration, Dundee, Scotland; 3 Garvan Institute of Medical Research, Darlinghurst, New South Wales, Australia; Duke University Medical Center, United States of America

## Abstract

**Background:**

Recent advances toward an effective therapy for prion diseases employ RNA interference to suppress PrP^C^ expression and subsequent prion neuropathology, exploiting the phenomenon that disease severity and progression correlate with host PrP^C^ expression levels. However, delivery of lentivirus encoding PrP shRNA has demonstrated only modest efficacy in vivo.

**Methodology/Principal Findings:**

Here we describe a new siRNA delivery system incorporating a small peptide that binds siRNA and acetylcholine receptors (AchRs), acting as a molecular messenger for delivery to neurons, and cationic liposomes that protect siRNA-peptide complexes from serum degradation.

**Conclusions/Significance:**

Liposome-siRNA-peptide complexes (LSPCs) delivered PrP siRNA specifically to AchR-expressing cells, suppressed PrP^C^ expression and eliminated PrP^RES^ formation in vitro. LSPCs injected intravenously into mice resisted serum degradation and delivered PrP siRNA throughout the brain to AchR and PrP^C^-expressing neurons. These data promote LSPCs as effective vehicles for delivery of PrP and other siRNAs specifically to neurons to treat prion and other neuropathological diseases.

## Introduction

The transmissible spongiform encephalopathies (TSEs), or prion diseases, are invariably fatal neurodegenerative disorders affecting primarily sheep (Scrapie), cattle (bovine spongiform encephalopathy), cervids (chronic wasting disease (CWD)) and humans (Creutzfeldt-Jakob and fatal familial insomnia). The prion hypothesis asserts that TSEs are caused by a misfolded, protease resistant isoform (PrP^RES^) of a cellular prion protein (PrP^C^) that is present on many mammalian cells but most highly expressed on neurons and glial cells in the central nervous system (CNS, [1], [2], [3]). Prions travel from peripheral infection sites to the CNS where they cause conformational changes in PrP^C^ to form PrP^RES^, and pathological changes in neural tissue including progressive neuronal cell death and gliosis [4], [5], [6], [7]. Presently, no effective therapies have been developed to treat TSEs.

An interesting aspect of prion diseases that researchers currently exploit as a therapeutic target is the necessity of PrP^C^ expression on CNS neurons for disease progression [8], [9], [10]. Decreasing neuronal PrP^C^ expression in scrapie-infected mice decreases incidence and severity, delays development [11], [12], [13] and can even reverse neuropathology of prion diseases [14]. The two most recent of these studies used RNA interference (RNAi), a relatively new tool for silencing specific genes in biological systems [15], [16], [17], [18] to decrease PrP^C^ within the CNS. RNAi has been shown to be potentially useful in treating several diseases including hepatitis [19], [20], cancer [21], ocular disorders [22] and chronic pain [23].

RNAi was first shown to effectively knock down PrP^C^ expression in cells transfected with PrP-encoding plasmids [24]. The same group later demonstrated efficient PrP^C^ knockdown in mice expressing an shRNA transgene, although they did not report whether these mice were protected from prion disease [25]. *In vivo* studies using intracranial (ic) injections of lentiviral vectors encoding PrP-specific shRNAs to knockdown focal PrP^C^ expression [12], [13] have shown modest effectiveness experimentally and limited use clinically due to the invasive delivery method, limited area and irreversibility of PrP^C^ suppression and safety concerns. More recently, Kumar et al have developed a transvascular method to deliver siRNA across the blood-brain barrier (BBB) to the brain via intravenous (iv) injection [26]. This method involves complexing siRNA to a short peptide derived from the rabies virus glycoprotein that binds specifically to acetylcholine receptors (AchRs) on neuronal cells [27], [28]. Adding nine d-Arginines to the carboxy terminus of this peptide (RVG-9r) enabled it to electrostatically interact with siRNA and specifically deliver siRNA to neurons in mouse brains to suppress protein expression and protect against fatal viral encephalitis. Efficient peptide-mediated delivery of siRNA to the brain after iv injection relies on protecting the complex from nuclease and protease degradation en route. Complexing or encapsulating siRNA with liposomes has been shown to protect siRNA from degradation and improve delivery through the vasculature [29], [30], [31], [32], [33], [34].

In the present study, we demonstrate the ability of cationic liposomes to protect siRNA from serum degradation and of the RVG-9r peptide to specifically target these liposome-siRNA-peptide complexes (LSPCs) to neuronal cells, knockdown PrP^C^ expression and greatly decrease PrP^RES^ in chronically prion-infected neuronal cells *in vitro*. Following injection of fluorescent RVG-9r LSPCs into mice, examination of brain sections indicated widespread presence of siRNA in the CNS. These data validate the use of LSPCs to protect siRNAs from serum degradation and deliver them across the BBB to AchR-expressing neuronal cells throughout the brain to potentially cure prion and perhaps other neurodegenerative diseases.

## Results

### Identification of effective PrP siRNA target sequences

**Figure 1:**
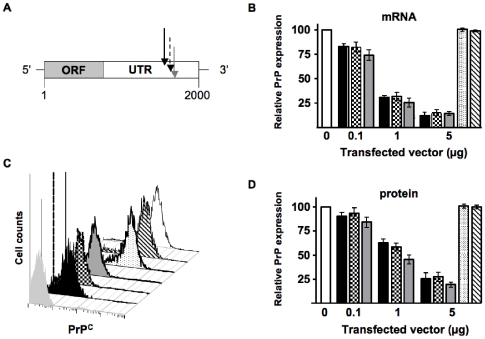
Transfection of shRNA plasmids effectively suppresses PrP^C^ mRNA transcript and protein levels in murine N2a cells. (A) Schematic showing the relative locations of the target sequences in the 3′ untranslated region of the murine *prnp* locus. Black, dotted and grey arrows designate target sequences of p1578, p1633 and p1672, respectively. (B) Quantitative RT-PCR shows the relative mRNA expression in N2a cells transfected with the indicated amounts of p1578 (black bars), p1633 (checkered), p1672 (grey), pControl (stippled) and empty vector (striped) relative to untransfected control cells (white). (C) FACS analysis of cell-surface PrP^C^ expression in transfected cells. Peak fill colors correspond to the bar colors shown in (B). The light grey peak represents unstained control cells. (D) Cumulative FACS data analysis. Bar colors correspond to the same sample groups shown in (B). All three shRNA plasmids reduced PrP^C^ transcript (B) and protein (C and D) expression in N2a cells in a dose-dependent manner. Data from each assay were collected from at least three independent experiments.

To determine target sequences of the *prnp* gene at which siRNA could effectively suppress PrP^C^ expression, we scanned approximately 1 kilobase of the 3′ untranslated region for siRNA targets predicted to reduce PrP transcript levels to less than 20% of normal [35]. We selected three sequences beginning at 1578, 1633 and 1672 bases after the transcriptional start site (table 1 and figure 1A), constructed plasmids encoding shRNAs targeting these sequences and a control sequence containing a scrambled 1672 sequence, transfected them into PrP-expressing N2a cells and analyzed their PrP expression 48 hours later. RT-PCR (figure 1B) and FACS analyses (figure 1C and D) demonstrate that all three shRNA plasmids suppressed PrP mRNA and protein levels, respectively, in a dose-dependent manner, to less than 25% of normal levels (p<0.01, n = 5). We detected no significant change in PrP mRNA and protein levels in N2a cells transfected with plasmids encoding no shRNA or scrambled 1672 sequence.

**Table 1:** PrP 3' UTR DNA target and delivery peptide sequences.

plasmid^1^	sequence^2^
p1578	GAAGTAGGCTCCATTCCAAA
p1633	GGCAAAATTTGTTCCTGAAG
p1672	ACATAAACTGCGATAGCTTC
pControl^3^	TCAAAATGTCGTCGTAAGAG
**peptide**	
RVG-9r^4^	YTIWMPENPRPGTPCDIFTNSRGKRASNGGGGrrrrrrrrr
RVM-9r^4^	MNLLRKIVKNRRDEDTQKSSPASAPLDGGGGrrrrrrrrr

1 numbers indicate position of first nucleotide relative to the *prnp* ORF start site.

2 DNA sequences read 5' to 3', protein sequences read N to C-terminus.

3 scrambled 1672 sequence.

4 lower case r denotes d-Arginine racemer.

### Liposome protection and peptide delivery of PrP siRNA to neuronal cells

**Figure 2:**
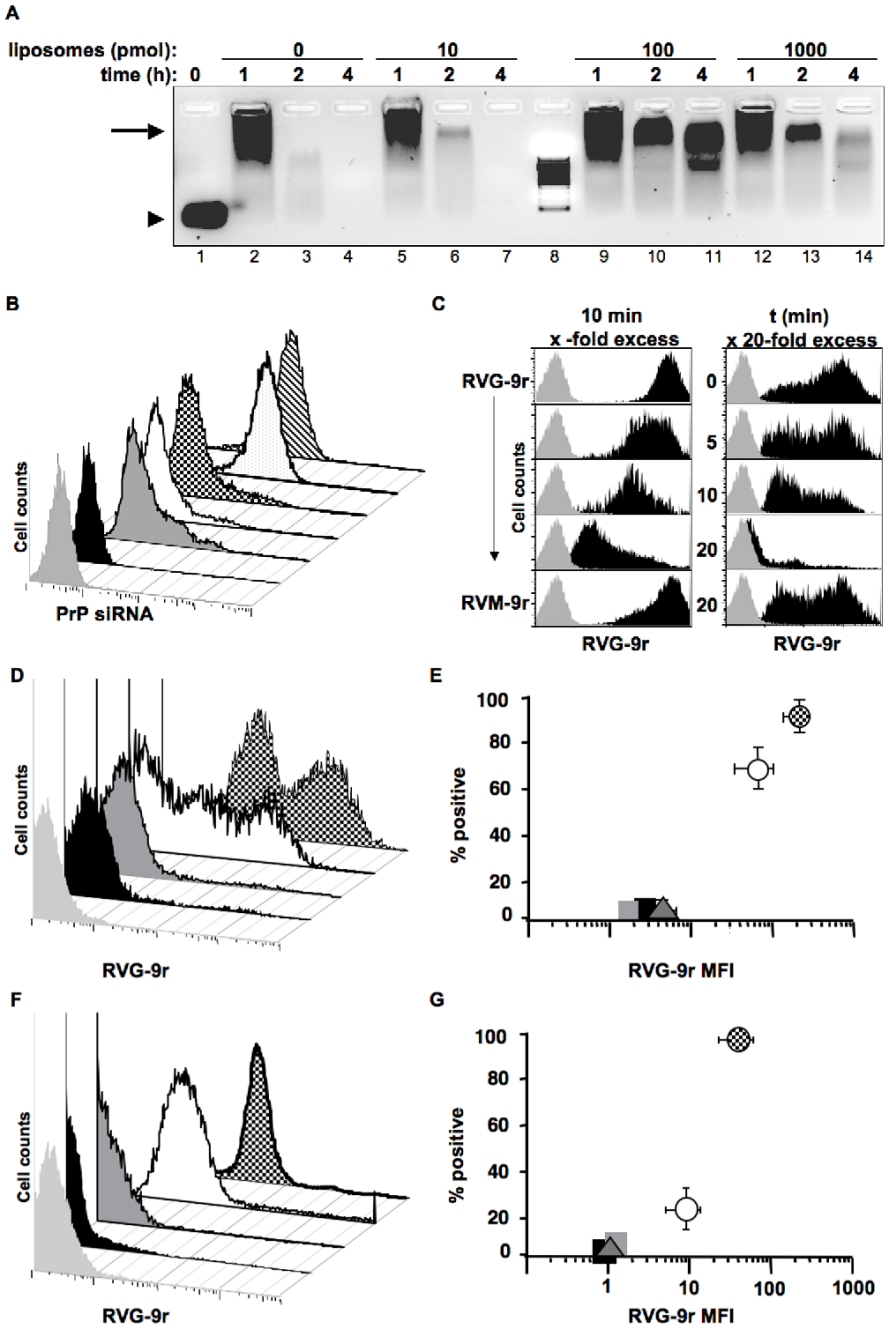
Liposome protection and peptide delivery of PrP siRNA to neuronal cells. (A) 100 pmol of PrP siRNA was incubated with the indicated amounts of liposomes then 1000 pmol of RVG-9r peptide. These siRNA-liposome-peptide complexes (LSPCs) were incubated at 37°C in the presence of 90% freshly isolated mouse serum for the indicated times then electrophoresed through a 2% agarose gel. The arrowhead indicates free siRNA and the arrow indicates siRNA-peptide complexes. Lane 8 contains an RNA molecular weight marker (bottom band is 100 bases). (B) 100 pmol of Alexa 488-labeled of PrP siRNA was incubated with 100 pmol of liposomes then 1000 pmol of RVG-9r or RVM-9r. LSPCs were added to N2a cells for four hours in the presence of media containing 10% FBS. Cells were harvested then analyzed by FACS. The black peak represents PrP siRNA complexed with control peptide RVM-9r in the absence of liposomes; dark grey peak, PrP siRNA-RVM-9r complex plus 1000 pmol liposomes; white peak, PrP siRNA-RVG-9r complex in the absence of liposomes; checkered peak, PrP siRNA-RVG-9r complexed with 10 pmol liposomes; stippled peak, PrP siRNA-RVG-9r with 100 pmol liposomes; striped peak, PrP siRNA-RVG-9r with 1000 pmol liposomes. (C) Competitive binding experiment using excess unlabeled RVG-9r or RVM-9r peptide against DyLight 488-labeled RVG-9r binding to N2a cells, varying either dose (left column) or time (right column) in the presence of 100 pmol liposomes. (D) Representative FACS analysis of DyLight 488-labeled RVG-9r-containing LSPCs binding to N2a (checkered peak), HEK (white peak), 4T1 (dark grey) or HeLa (black) cells. (E) Cumulative data analysis of RVG-9r LSPCs binding to N2a (checkered circle), HEK293 (white circle), 4T1 (dark grey triangle) and HeLa (black square) cells. Representative FACS (F) and cumulative data (G) analyses of RVG-9r LSPCs binding to brain (checkered peak (F) and circle (G)) and kidney (white peak and circle) cells, splenocytes (dark grey peak and triangle) and hepatocytes (black peak and square). The light grey peaks in B, C, D, and F indicate unstained controls.

A 29-residue peptide from the rabies virus glycoprotein has been previously shown to bind acetylcholine receptors (AchRs) with high affinity and specificity [28]. Addition of nine d-Arginine residues enabled the peptide, RVG-9r, to bind siRNA and deliver it specifically to neuronal cells after iv injection to suppress protein expression and cure Japanese Encephalitis viral infection [26]. We sought to improve RVG-9r delivery of siRNA by protecting siRNA-peptide complexes from serum degradation by adding liposomes. One hundred picomoles of siRNA were complexed with various concentrations of sonicated and filtered liposomes then with 1000 pmol of RVG-9r. The resulting liposome-siRNA-peptide complexes (LSPCs) were 178±20 nm in hydrodynamic diameter with surface charges ranging from 6.25±0.75 to 9.78±0.96 to 19.89±4.46 mV with 10, 100 and 1000 pmol of liposomes, respectively. LSPCs were incubated in 90% mouse serum at 37°C and assayed for degradation over time. Addition of 100 pmol of liposomes prevented significant serum degradation of siRNA and peptide for at least four hours (figure 2A). To determine whether RVG-9r could deliver siRNA specifically to AchR-expressing neuronal cells when complexed with liposomes, we exposed LSPCs composed of either DyLight 488-labeled RVG-9r or control RVM-9r peptide and increasing concentrations of liposomes to N2a cells in the presence of 10% fetal bovine serum (FBS). Control peptide RVM-9r in the absence of liposomes failed to deliver PrP siRNA to N2a cells, while 1000 pmol of liposomes in the PrP siRNA-RVM-9r complex facilitated siRNA delivery to approximately 15% of N2a cells (figure 2B). RVG-9r peptide complexed to PrP siRNA in the absence of liposomes delivered siRNA to 8% of N2a cells, but increased to 12% when complexed with 10 pmol liposomes, and to over 85% when complexed with 100 pmol or more of liposomes. We used equimolar concentrations of siRNA and liposomes in the remainder of the experiments because this ratio produced the highest cell specificity and protection from serum degradation. Molar excesses of unlabeled RVG-9r, but not controlRVM-9r, successfully competed against fluorochrome-tagged RVG-9r for AchR binding in a time and dose dependent manner, confirming RVG-9r specificity (figure 2C).

To confirm cell specificity, Alexa 488-labeled PrP siRNA-RVG-9r-containing LSPCs were incubated with several cell lines that were then assayed for LSPC delivery by FACS. LSPCs were delivered to 90±5% of N2a cells with a mean fluorescence intensity (MFI) of 296±44, significantly more than to 67±7% of human embryonic kidney 293 cells (HEK 293 cells, figures 2D and E, p = 0.03) with a MFI of 74±21 (p<0.01). LSPCs bound to less than 5% of 4T1 breast cell carcinoma or HeLa cervical cell lines with MFIs below 10, significantly less than either N2a or HEK 293 cells (p<0.01, n = 5). Similarly, RVG-9r-containing LSPCs bound to 98±1% of brain cells with a MFI of 82±5, significantly more than to 23±11% of kidney cells (figures 2F and G, p<0.01) with a MFI of 11±4 (p<0.01). RVG-9r LSPCs bound to less than 2% of splenocytes and hepatocytes with MFIs approximating 1, significantly less than either brain or kidney cells (p<0.01, n = 3).

### PrP siRNA-RVG-9r LSPC delivery kinetics

**Figure 3:**
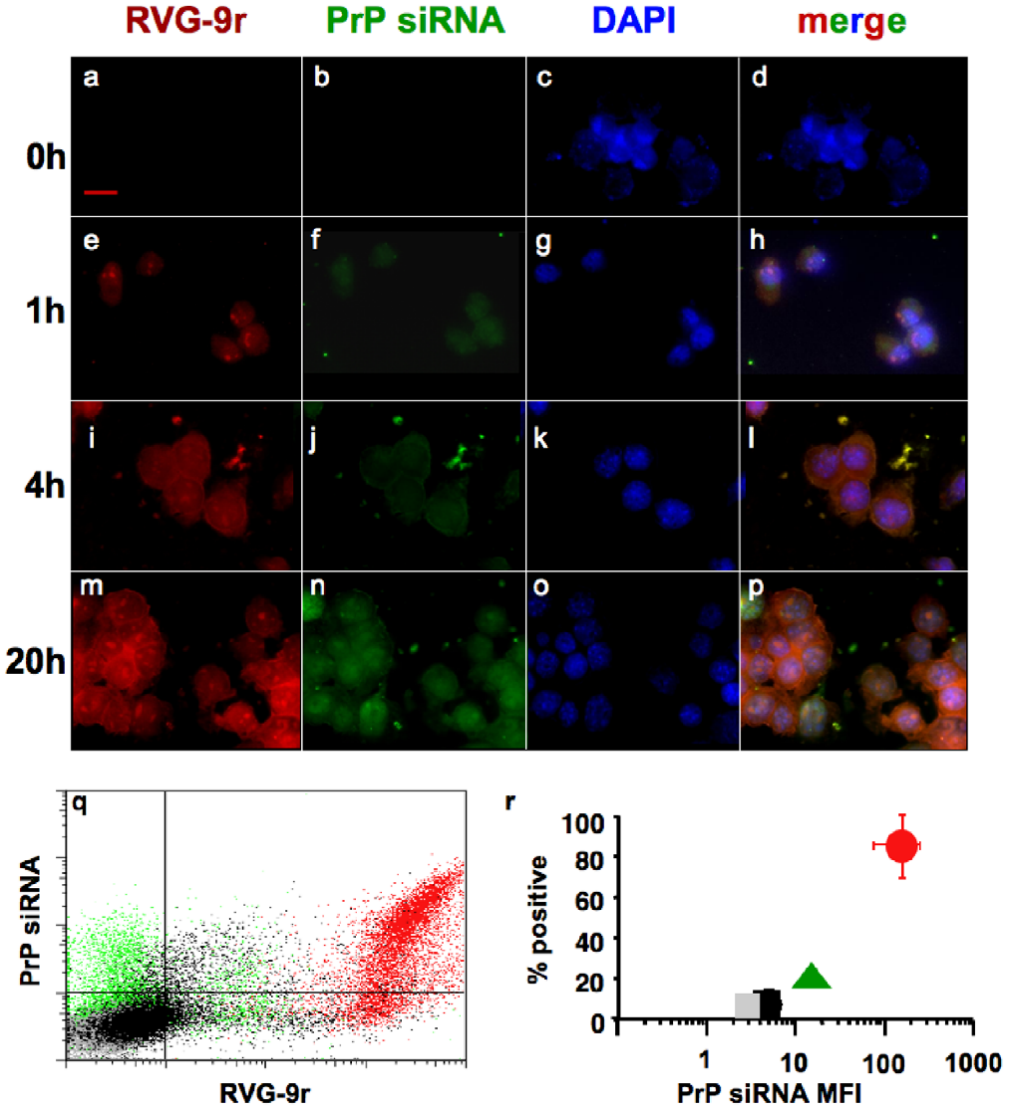
PrP siRNA-RVG-9r LSPC delivery kinetics. N2a cells were visualized before (panels a–d) or 1 h (e–h), 4 h (i–l) and 20 h (m–p) after incubation with LSPCs containing DyLight 649-labeled RVG-9r (a, e, i and m) and Alexa 488-labeled PrP siRNA (b, f, j and n). Cell nuclei were visualized with DAPI (c, g, k and o). (q) FACS analysis at 24 h of N2a cells treated with LSPCs containing PrP siRNA and RVG-9r (red dots) or control RVM-9r (black) peptides or liposomes alone (green). Grey dots represent untreated cells. (r) Cumulative data from five separate delivery experiments. The red circle represents data from N2a cells treated with RVG-9r and PrP siRNA-containing LSPCs; black square, PrP-RVM-9r LSPCs; green triangle, liposomes alone. Grey square, untreated cells.

We monitored LSPC delivery to N2a cells over time by fluorescent microscopy and FACS analyses to better understand LSPC delivery kinetics. Merged color images reveal LSPC deposition on cells beginning 1 h after incubation (figure 3, compare panels a–d to e–h) that appears increased 4 h later (i–l), with marked cell surface localization (l). Color separation indicates LSPC dissociation at 20 h (m–p), with PrP siRNA appearing more perinuclear, where mRNA silencing occurs [36]. FACS analysis at 24 h indicates LSPCs containing RVG-9r peptide substantially increased siRNA delivery to N2a cells over LSPCs containing control RVM-9r peptide or liposomes alone (figure 3q). Cumulative data from five separate experiments shows RVG-9r peptide delivered PrP siRNA to 85±10% of N2a cells with a MFI of 200±31 (figure 3r), significantly more than RVM-9r peptide (7±4% with an MFI of 5±3) or liposome alone (20±4% with an MFI of 10±2, p<0.01), values not significantly different than untreated cells (grey square, 3±2%, MFI of 3±2).

### PrP-RVG-9r LSPCs suppress PrP^C^ expression

**Figure 4:**
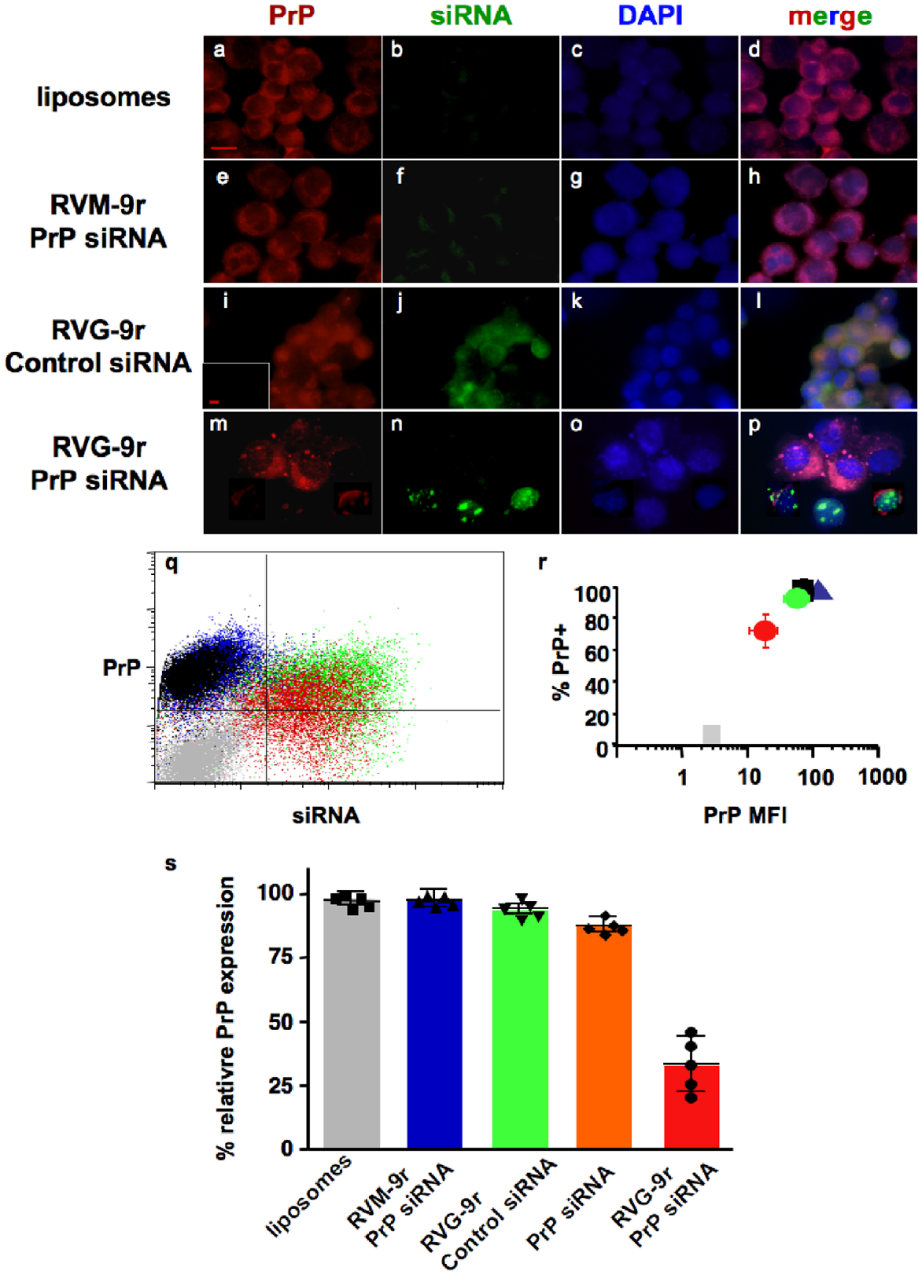
PrP LSPCs suppress PrP expression. N2a cells treated with liposomes alone (panels a–d), or LSPCs containing Alexa 488-labeled siRNAs, including RVM-9r control peptide and PrP siRNA (e–h), RVG-9r peptide and control siRNA (i–l) or RVG-9r and PrP siRNA (m–p). Cells were harvested 48 hours later and stained with DyLight 649-labeled anti-PrP antibody (panels a, e, i and m). Cell nuclei were visualized with DAPI (c, g, k and o). Inset in panel (i) depicts a serial section stained with an irrelevant isotype control antibody. (q) FACS analysis of N2a cells treated with PrP-RVG-9r (red dots), PrP-RVM-9r (blue), or RVG-9r-control siRNA (green) LSPCs or liposomes alone (black). Grey dots represent untreated, unstained N2a cells. (r) Quantification of data from at least five independent experiments. Red circle represents N2a treated with PrP-RVG-9r LSPCs; green circle, control- RVG-9r LSPCs; blue triangle, PrP-RVM-9r LSPCs; black square, liposomes alone; grey square, untreated and unstained N2a cells. (s) Cumulative data comparing relative PrP^C^ expression on N2a cells treated with liposomes alone (grey bar), PrP-RVM-9r (blue), control-RVG-9r (green) or PrP-RVG-9r (red) LSPCs or PrP siRNA and liposomes only (orange). Symbols associated with each bar indicate values for five independent experiments.

We next investigated whether PrP LSPCs could effectively suppress PrP^C^ expression. LSPCs were formed and exposed to N2a cells, which were analyzed for siRNA delivery and PrP^C^ expression 48 h later. LSPCs containing RVG-9r (figures 4i–l), but not RVM-9r (figures 4e–h) or liposomes alone (figures 4a–d), delivered siRNA that could be visualized directly by fluorescent microscopy. Merged images (figures 4d, h, l and p) reveal substantial PrP^C^ expression in all treatment groups except N2a cells treated with RVG-9r-PrP siRNA-containing LSPCs, where PrP^C^ expression appeared significantly diminished, especially on cells with Alexa 488-labeled PrP siRNA still evident (figures 4m–p).FACS analysis of treated cells indicates reduced expression of PrP^C^ on cells treated with PrP-RVG-9r LSPCs, with little or no change in PrP^C^ expression on cells treated with PrP-RVM-9r LSPCs, control-RVG-9r LSPCs or liposomes alone (figure 4q). Quantification of data from at least five independent experiments (figure 4r) revealed that PrP-RVG-9r LSPCs significantly decreased both the proportion of PrP^C^-positive cells (70±9%) and the PrP MFI (20±7) on N2a cells (p<0.01). All other groups expressed PrP^C^ on greater than 85% of cells with MFIs approximating 100. Cumulative data analysis shows that PrP-RVG-9r LSPCs significantly decreased PrP^C^ expression on N2a cells to 30±8% (figure 4s, p<0.01) relative to N2a cells treated with PrP-RVM-9r LSPCs (99±3%), RVG-9r-control LSPCs (98±2%) or liposomes alone (99±2%) or containing PrP siRNA without peptides (88±5%). While RVG-9r LSPCs efficiently delivered PrP siRNA to HEK293 cells, they failed to suppress PrP^C^ expression (data not shown). The 3′ UTR in the human *PRNP* gene does not contain the PrP siRNA target sequence, and HEK 293 cells thus serve as an additional negative control for PrP siRNA-specific suppression of murine PrP^C^. Taken together, these data demonstrate effective delivery of siRNA to N2a cells using RVG-9r LSPCs and specific suppression of murine PrP^C^ expression when these LSPCs delivered PrP siRNA.

### PrP LSPCs eliminate PrP^RES^ in vitro

**Figure 5:**
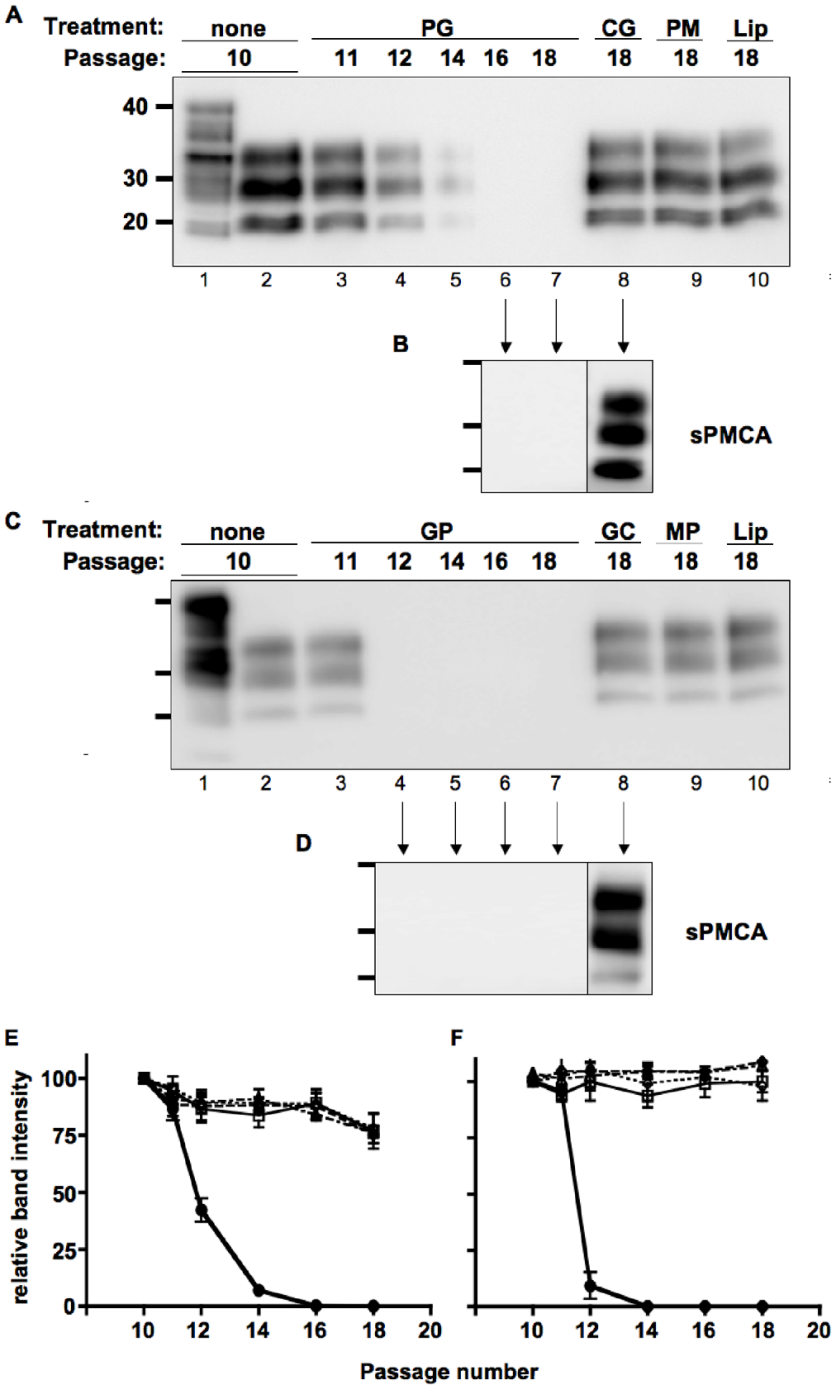
PrP LSPCs cure prion-infected cells in vitro. Chronically-infected N2a (A and B) and NCerPrP (C and D) cells were treated for 72 h with PrP-RVG-9r (PG, lanes 3–7), control-RVG-9r (CG, lane 8), or PrP-RVM-9r LSPCs (PM, lane 9) or liposomes alone (Lip, lane 10). Cells were split at the indicated passage and excess cells pelleted, digested with 10 mg/mL proteinase K, electrophoresed and immunoblotted for PrP^RES^. Negative samples, and one positive sample diluted 1000-fold, from each infection were subjected to six 48-cycle rounds of PMCA (B and D). Molecular weight markers are shown for 20, 30 and 40 kilodaltons to the left of each blot. (E and F) Densitometric analyses of western blots from three independent experiments treating prion infection in both N2a (E) and NCerPrP (F) cells. Black circles connected by solid lines represent cells treated with PrP-RVG-9r LSPCs; open squares connected by solid lines, Control-RVG-9r LSPCs; black squares connected by dashed lines, PrP-RVM-9r LSPCs; and black triangles connected by dotted lines, Liposomes alone.

To ascertain whether LSPC knockdown of PrP^C^ expression sufficed to cure prion infection, we used two cell culture models of prion infection. N2a and NCerP cells were infected with the Rocky Mountain Lab strain of mouse-adapted scrapie prions (RML) and E2 CWD prions, respectively, and highly susceptible clones isolated, expanded, and aliquots frozen as previously described [37]. Prion-infected N2a and NCerP cells were passaged ten times, at which time they were treated with LSPCs one time then grown to confluency and passaged an additional eight times. Cells from each treatment group were collected at each passage and assayed for PrP^RES^. We detected no scrapie PrP^RES^ (PrP^Sc^) in RML-infected cells treated with PrP-RVG-9r LSPCs after passage 14 (figures 5A and E). We failed to detect even minute amounts of residual PrP^Sc^ in these cells through an ultrasensitive prion amplification technique called serial protein misfolding cyclic amplification (sPMCA, Figure 5B [38], [39]), while we detected significant PrP^Sc^ in control-treated cells at passage 18 with or without sPMCA. Similarly, we detected no CWD PrP^RES^ (PrP^CWD^) in CWD-infected cells treated with PrP-RVG-9r siRNA after passage 12, while we detected PrP^CWD^ in control-treated cells as far as passage 18 (figures 5C, D and F). Previous studies on these prion strains revealed that PMCA can amplify serial dilutions of RML and E2 two to three orders of magnitude per round ([38], [40] and our unpublished data), or a 10^−12^ to 10^−18^-fold dilution after six PMCA rounds. These data convincingly demonstrate that PrP LSPCs can sufficiently knockdown PrP^C^ expression to decrease prion infection beyond delectability in two distinct cell culture models.

### LSPC delivery to PrP^C^-expressing cells in vivo

**Figure 6:**
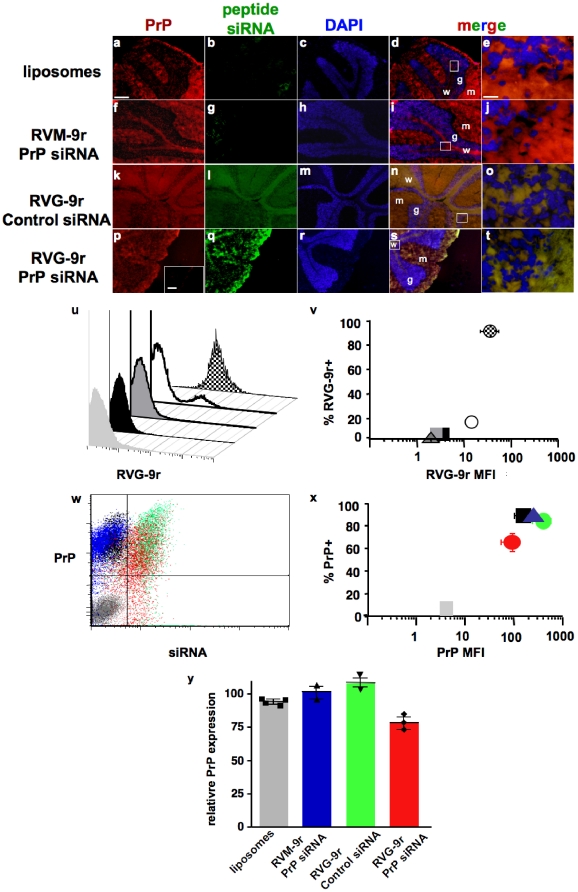
LSPC delivery to PrP^C^-expressing cells in vivo. FVB mice were intravenously injected with liposomes alone (panels a–e) or LSPCs containing DyLight 488-labeled peptides and Alexa 488-labeled siRNAs as follows: PrP-RVM-9r (f–j), Control-RVG-9r (k–o), or PrP-RVG-9r (p–t). Mice were sacrificed 24 hours later, dissected, and their brains snap frozen and 8 µm sections thereof stained with DyLight 649-labeled anti-PrP antibody (a, f, k and p) and DAPI (c, h, m and r) and visualized by fluorescence microscopy. Panels d, f, m and s are labeled to show the granular and (g) molecular (m) layers and white matter tracts (w), Panels e, j, o and t depict magnifications of the granular layer and white matter boundary in the boxed areas in panels d, i, n and s. Inset in panel p depicts a serial section stained with an irrelevant isotype control antibody. Scale bars, 50 µm for all panels except e, j, o and t, which is 10 µm. (u and v) FACS analysis of PrP-RVG-9r LSPC delivery to brain cells (checkered peak (u) and circle (v)), kidney cells (white peak and circle), splenocytes (dark grey peak and triangle) and hepatocytes (black peak and square). The light grey peak (u) and square (v) depict PBS-treated, unstained control brain cells. (w and x) FACS analysis of PrP^C^ expression on brain cells treated with PrP-RVG-9r (red dots (w) and circle (x)), RVG-9r-control (green dots and circle), PrP-RVM-9r (blue dots and triangle) LSPCs, or liposomes alone (black dos and square). The grey dots and square depict untreated, unstained brain cells. (y) Cumulative data showing relative PrP expression on brain cells treated with liposomes alone (grey bar) or with LSPCs containing PrP-RVM-9r (blue), control-RVG-9r (green) or PrP-RVG-9r (red). Symbols associated with each bar indicate values for individual mice.

Finally, we assessed the ability of RVG-9r LSPCs to protect and deliver siRNA specifically to neuronal cells in live animals. LSPCs containing 4 nmol of siRNA and liposomes and 40 nmol of peptide were injected into the tail veins of FVB mice and LSPC delivery assayed 24 hours later by fluorescent microscopy and FACS. Merged color images reveal strong PrP staining in the cerebellum, where significant prion neuropathology has been previously documented [38], [41]. PrP staining was particularly strong in white matter tracts and the molecular layer but also evident in the granular layer (Figures 6d, e, i, j, n, o, s and t). Liposomes alone (figures 6a–e) or the RVM-9r peptide (figures 6f–j)delivered little or no siRNA, while the RVG-9r peptide delivered easily detectable amounts of siRNA to areas that appear to overlap with PrP^C^ expression (figures 6k–t). FACS analyses demonstrate that RVG-9r delivered siRNA to 93±3% of brain cells with a MFI of 40±18 (figure 6u and v), to significantly less kidney cells (16±3% with a MFI of 16±5), splenocytes and hepatocytes (both less than 3%, with MFIs less than 3, p<0.01, n = 5), approximating values for liposome-only controls (2±1%, MFI = 2.5±1, n = 4). RVG-9r delivery of PrP siRNA correlated with reduced numbers of PrP-expressing brain cells (figure 6w and x, 63±9%) and PrP MFI (90±27, n = 3) compared to cells treated with liposomes alone (95±2%, MFI = 150±37), RVM-9r- PrP siRNA (95±5%, MFI = 202±19, n = 2) and RVG-9r-control siRNA (84±7%, MFI = 369±77, n = 2). Somewhat surprisingly, after just 24 hours, brain cells from PrP-RVG-9r LSPC-treated mice (n = 3) reduced PrP^C^ expression to approximately 75±11% (figure 6y) relative to all control groups combined (p<0.05, n = 8). These data indicate specific delivery of significant amounts of PrP siRNA to PrP^C^-expressing cells using RVG-9r LSPCs.

## Discussion

The efficacy of reducing PrP^C^ expression to delay or even reduce prion disease and neuropathology has been established [11], [12], [13], [14]. Several recent studies have demonstrated the potential of using PrP-specific shRNAs encoded by lentiviral constructs injected ic to knock down PrP^C^ expression [12], [13]. Several aspects of this method limit its efficacy. Poor transport across the BBB dictates delivery by stereotactic injection of PrP shRNA lentivirus directly into the brain. Even then, viral infection and shRNA expression was limited to a small area around the injection site, resulting in spatially limited suppression of PrP^C^ expression, modestly impeded disease progression and unaltered disease incidence. This method also lacks desired temporal control of PrP^C^ expression, since once brain cells are infected with lentivirus, it will likely irreversibly suppress PrP^C^ expression in these cells. Moreover, despite improvements in lentiviral vector technology and construction [42], [43], [44], [45], [46], concerns over the oncogenic capacity of lentiviral delivery systems remain [47]. Another group created transgenic mice stably expressing PrP shRNA that reduced PrP^C^ expression five-fold, although they did not assess prion disease in these animals [25]. This method demonstrates an important proof-of-principle, but suffers from the same irreversibility of PrP^C^ suppression as lentiviral shRNA delivery, and transgenesis is not a viable therapeutic option.

In this study, we have begun to explore the possibility of using LSPCs injected iv to deliver PrP^C^ siRNA across the BBB to all areas of the brain to suppress PrP^C^ expression on neurons to protect them from prion diseases. RVG-9r, a modified peptide from the rabies virus glycoprotein that acts as a ligand for AchRs and whose positively charged Arginine tail binds the negatively charged phosphate backbone of siRNAs, has been shown to effectively target siRNA to neuronal cells when delivered through the vascular system to the CNS [26]. We postulated that RVG-9r could act as a molecular address for PrP siRNA as well and used it as such in experiments described here.

In order for siRNA injections to be useful in treating prion diseases, serum degradation must be prevented during vascular transport. Kumar reported only partial protection of siRNA by RVG-9r from serum degradation. Zimmerman found more than a 100-fold improvement in gene silencing when siRNA was complexed to lipids [34]. Other studies have also shown that serum half-life is greatly improved when siRNA is encapsulated within a liposomal nanoparticle [23], [32]. Taken together, these studies indicate that liposomes increase the stability of the siRNA-peptide complex in serum leading to increased treatment efficacy. After identifying suitable RNAi targets in the 3′ UTR of the *prnp* gene and synthesizing cognate siRNAs, we tested the ability of cationic liposomes to protect them and the RVG-9r peptide from serum degradation. One hundred pmol of liposomes maximally protected LSPCs from degradation after 4 hours of incubation in mouse serum. We also found that LSPCs increased the siRNA binding to N2a cells in a dose-dependent manner, presumably by protecting siRNA-RVG-9r complexes, thereby increasing their bioavailability. Interestingly, 1000 pmol of liposomes decreased LSPC protection and specificity in our assay. Perhaps excess liposomes encapsulated protease and nuclease-laden serum and fused to LSPCS, degrading them over time, Indeed, 1000 pmol of liposomes nonspecifically delivered LSPCS to cells, indicating that excess liposomes may be fusing with cell membranes, supporting this interpretation. We therefore used equimolar amounts of liposomes and siRNA for subsequent experiments.

Our *in vitro* experiments show the specificity of RVG-9r LSPCs for N2a and HEK 293 cell lines, as well as primary neuronal cells and a small proportion of primary kidney cells, but not to HeLa or 4T1 breast carcinoma cell lines or primary splenocytes or hepatocytes. Control RVM-9r LSPCs did not bind to any of these cell types. HEK 293 cells, created by transformation of primary HEK cells with the preferentially neurotropic Ad5 virus, express many neuronal proteins, including AchRs, and are likely of neuronal lineage [48], [49]. The inner medullary collecting duct cells of the kidney also express AchRs [50], [51]. These LSPC binding results expand on those demonstrating RVG binding to N2a cells, but not HeLa cells or 293T cells, created by transformation of HEK cells with the SV40 T antigen, and distinct from the HEK 293 cells that we used [49]. Both HeLa and 293T cells have been shown to lack AchRs[26], and several microarray analyses have failed to demonstrate AchR expression in 4T1 cells [52], [53], [54], consistent with lack of RVG binding to them. RVG-9r LSPCs were delivered to AchR-expressing cells in as little as one hour, with LSPC dissociation and siRNA movement to the perinuclear space by twenty hours after treatment.

We further show that AchR-expressing neuronal cells specifically targeted by RVG-9r LSPCs also express PrP^C^, which was suppressed 70% by PrP siRNA LSPCs 48 hours after treatment. Reduced, punctate staining of cell-surface PrP^C^ suggests an impaired ability to replace older PrP^C^ being removed by normal cell membrane turnover and/or protein recycling with nascent PrP^C^, whose synthesis is greatly inhibited by LSPCs.

A single treatment with PrP siRNA-RVG-9r LSPCs decreased the amount of PrP^SC^ and PrP^CWD^ formation to undetectable levels in chronically prion-infected infected cultures of N2a and NCerP cells, respectively, while infected cultures receiving control LSPCs still contained PrP^Sc^ after 18 passages. We also subjected cured cell lysates to six rounds of serial PMCA and detected no PrP^RES^. We have successfully detected up to a 10^−18^-fold dilution of RML and E2 prions using PMCA, which is far more sensitive than N2a cell culture assays, which can replicate PrP^Sc^ from RML dilutions up to 10^−8^ to sustain prion infection [37], [55], [56]. We therefore conclude that PrP siRNA-RVG-9r LSPCs dramatically decreased prion infection beyond sustainable levels in two cell culture models.

Kim et al recently reported that stable transfection of PrP shRNA plasmids into ScN2a cells chronically infected with Syrian hamster prions [57] initially cleared prion infection below detection but failed to clear all PrP^Sc^, as evidenced by a recurrence of PrP^Sc^ by further passage or PMCA of cell pellets [58]. Differences in prion strains, infection protocols and host cell clones may account for differences among these three studies. Importantly, though, RNAi treatment in all of these model systems dramatically impeded PrP^RES^ replication by reducing PrP^C^ expression by more than 50%, an expression threshold shown to dramatically delay disease onset [11], [12], [13], [14]. Genetic or siRNA-mediated PrP^C^ depletion in these studies delayed or even prevented disease, while still replicating PrP^Sc^ to levels seen in wild type mice expressing normal levels of PrP^C^. Therefore, the presence of PrP^RES^ may not reliably predict disease incidence and severity in vivo. Transient, partial PrP^C^ knockdown may sufficiently arrest disease progression such that treated patients may live normal life spans with reduced pathology and clinical symptoms, albeit with potentially significant PrP^RES^ accumulation.

RNAi therapy using lentiviral delivery exhibited marginal efficacy in ameliorating disease and pathology in previous in vivo prion disease models, most likely because direct ic injection limited viral transduction to cells immediately adjacent to the injection site [12], [13]. We sought to improve PrP siRNA delivery via iv administration of LSPCs. Many cells in the brain that are important in prion pathogenesis express AChRs, including neurons, astrocytes and microglia [59]. Following iv injection of fluorescent RVG-9r LSPCs into FVB mice, we found many labeled cells throughout the brain that also expressed PrP^C^, indicating that PrP siRNA can be delivered across the BBB to PrP^C^-expressing neuronal cells by a AchR-binding peptide.

A previous study demonstrated transvascular delivery of GFP-labeled siRNA to the CNS using the RVG-9r peptide alone [26]. However, FITC-labeled siRNA was directly visible in only a fraction of the brains from treated mice, with most requiring an additional anti-FITC staining to visualize the siRNA. We detected fluorescent siRNA in all mice treated with RVG-9r LSPCs without a secondary stain, suggesting that LSPCs protected siRNA-peptide complexes from significant serum degradation, facilitating delivery of more siRNA that was directly visible in the brain 24 hours after iv injection. However, we also used fluorescent RVG peptide emitting at the same wavelength as PrP siRNA to enhance detection that was problematic in previous studies. So using both 488 nm-fluorescent peptide and RNA likely contributed to our increased sensitivity. In either case, our study demonstrates effective delivery of siRNA to the brain using LSPCs.

While we designed this experiment to test RVG-9r LSPC-mediated PrP siRNA delivery to PrP^C^-expressing neurons, we also observed a significant decrease in PrP^C^ expression on CNS cells after just 24 hours post treatment. We detected insignificant amounts of RVG-9r LPSCs binding to non-neuronal cells, which is important in both focusing treatment and limiting potential side effects of siRNAs. These experiments build on previous ones demonstrating GFP and SOD1 gene silencing only in the brain following iv administration of RVG-9R siRNA to GFP transgenic and wild type Balb/c mice, respectively [26]. Intravenous treatment with siFvE + RVG-9R was also able to protect 80% of inoculated mice from fatal flaviviral encephalitis. The RVG-9r peptide elicited no adverse inflammatory or other immune response in treated mice, which exhibited no adverse side effects and appeared to tolerate the treatment very well. Our system involves the addition of cationic liposomes to siRNA-RVG-9r complexes. Cationic liposomes carrying plasmid or CpG DNA have been used as adjuvants to specifically enhance immunity against infection and tumors [60], [61], . We carefully screened siRNA sequences to specifically avoid the Interferon response [64], [65] and used liposome concentrations well below those used as adjuvants to prevent immunogenicity. We also constructed LSPCs fifty times smaller than microparticles shown to become trapped in lung tissue [66]. Indeed, we saw no evidence of LSPC uptake by immune cells such as macrophages and dendritic cells in the spleen, and observed no evidence of lung pathology in treated mice. However, further in vivo studies need to confirm that LSPCs do not elicit potentially harmful immune responses.

We envision LSPC therapy to be a new delivery system that greatly enhances the capacity of PrP siRNA to transiently suppress PrP^C^ expression in nearly every neuron in the CNS that will significantly impede prion replication and disease progression. Whether therapy can rescue patients already exhibiting clinical signs of prion disease must be determined. The most effective therapy, however, will be coupled with improved diagnostic techniques that allow the earliest possible detection of prion diseases, including early behavioral and cognitive tests currently being developed [13], [67], [68].

LSPC therapy targeting host protein expression may also benefit other protein misfolding disorders. For example, PrP^C^ has been shown to bind Amyloid-β oligomers and mediate synaptic dysfunction in hippocampal slice cultures, suggesting a potential role for PrP^C^ in Alzheimer's disease [69]. LSPCs targeting PrP^C^, perhaps in combination with Amyloid Precursor protein expression may prove to be a potent therapy for a disease that affects millions of people worldwide.

## Methods

### Mice

FVB mice were purchased from Charles River (Wilmington, MA) and maintained and handled in strict accordance with good animal practice as defined by relevant national and/or local animal welfare bodies, and all animal work was approved by Colorado State University Institutional Animal and Care Use Committee (IACUC approval numbers 08-146ABC-02 and 08-281A-02).

### Cell lines and culture

N2a, HEK 293, HeLa and 4T1 cells were purchased from ATCC (Manassas, VA). NCerP cells are a derivation of N2a cells that express cervid PrP^C^ instead of mouse PrP^C^ (our unpublished data). Briefly, we enriched for N2a cells lacking endogenous PrP^C^ expression (NΔ cells) by five rounds of fluorescence activated cell sorting (FACS) and clonal expansion. Lipofectamine 2000 reagent was used according to manufacturer's protocol (Invitrogen) to transfect NΔ cells with a 1∶10 ratio of a plasmid encoding the neomycin resistance gene and the PrP half genomic construct [70] in which the murine PrP ORF was replaced by the cervid PrP ORF from mule deer. G418-resistant clones were selected and cervid PrP expression confirmed by FACS (data not shown). Cell lines were maintained in RPMI media supplemented with 10% fetal bovine serum (FBS), 100 U/mL Penicillin and 100 µg/mL Streptomycin (1X Pen-Strep, GIBCO). Primary cells were harvested from brain, spleen, liver and kidney of FVB mice and passed through a 45 µm nylon mesh to create single cell suspensions in RPMI, 10% FBS, 1X Pen-Strep.

### RNAs

PrP 3′ untranslated region (UTR) DNA target and control sequences (table 1, GenBank accession number NM_011170) were identified using siRNA scales [35]. Plasmids encoding shRNA targeting these sequences were constructed using oligonucleotides synthesized, purified, annealed and cloned first into the pENTR/D-TOPO plasmid and then subcloned into the pBLOCK-iT 3-DEST Vector plasmid using the Gateway cloning system according to the manufacturer's protocol (Invitrogen, Carlsbad, CA). A 4-base 5′-CGAA-3′ loop was inserted between the complimentary sequences encoding the shRNAs. Duplex siRNAs targeting the same sequences and AllStars negative control siRNAs were synthesized, purified, annealed and resuspended in RNase-free water and, where indicated, labeled with Alexa 488 on the 5′ sense strand (Qiagen, Valencia, CA).

### Peptides and liposomes

Peptides RVG-9r and RVM-9r (table 1) were previously described [26] and synthesized, purified by high performance liquid chromatography (Pi Proteomics, Huntsville, AL) and resuspended in sterile 1X PBS. Where indicated, peptides were labeled with DyLight 488 or 649 fluorochromes according to manufacturer's protocol (Pierce, Rockford, IL). Liposomes were prepared as previously described [63] with slight modifications. Briefly, equimolar concentrations of the cationic lipid octadecenolyoxy[ethyl-2-heptadecenyl-3 hydroxyethyl] imidazolinium chloride (DOTIM, Sigma-Aldrich, St. Louis, MO) and cholesterol (Avanti Polar Lipids, Alabaster, AL) in chloroform were added to round-bottom, 15-mL glass tubes to a final concentration of 2 mM. The solution was then dried overnight in a vacuum desiccator to a thin film. Liposomes were rehydrated to a final concentration of 10 mM in 10% sucrose in water at 50°C for 50 min, followed by incubation for 2 h at room temperature and serially filtered through 1, 0.45 and 0.2 µM pore filters (Pall Life Sciences, Port Washington NY) to achieve uniform size and sterility then stored under argon at 4°C. Liposomes were sonicated 3×40 s at 70% maximum power in a MP4000 horn sonicator (Misonix) then diluted to working concentrations immediately prior to use.

### Plasmid transfection and PrP^C^ mRNA and protein analyses

shRNA plasmids were transfected into 10^5^ N2a cells at the given concentrations using Lipofectamine 2000 transfection reagent (Invitrogen). Cells were harvested 48 hours later and analyzed for PrP^C^ mRNA and protein expression. mRNA was isolated using the Oligotex Direct mRNA mini kit reverse-transcription polymerase chain reaction (RT-PCR) performed in real-time using QuantiTect SYBR Green RT-PCR Kit (Qiagen). PrP transcripts were amplified in an iCycler RT-PCR machine (Bio-Rad, Hercules, CA) using the forward primer 5′- CCT TGG TGG CTA CAT GCT GG-3′ and reverse primer 5′-GGC CTG TAG TAC ACT TGG-3′. DNA templates were pre-melted at 95°C for five minutes; then amplified for thirty cycles of melting at 95°C for one minute, primer annealing at 50°C for thirty seconds and amplicon elongation at 72°C for one minute; followed by one final elongation cycle at 72°C for five minutes. Cycle threshold values were determined automatically by iCycler software and normalized by those for β-actin transcripts amplified using the Mm_Actb_2_SG QuantiTect Primer Assay (Qiagen). Amplicon specificity was determined by melting curve analysis and confirmed by agarose gel electrophoresis (data not shown). PrP mRNA expression was reported relative to that for untreated N2a cells.

### Fluorescence Activated Cell Sorting (FACS)

For protein analysis, 10^6^ transfected and control N2a cells were washed three times with FACS buffer (10 mM EDTA, 0.1% fetal bovine serum in 1X PBS), incubated for ten minutes in 10% mouse serum in FACS buffer, stained with 1∶100 dilution of DyLight 649-labeled Bar224 (SPI Bio) in 100 µl of FACS buffer supplemented with 5% mouse serum for thirty minutes, washed three times with FACS buffer, resuspended in 1 mL FACS buffer and analyzed using a Cyan multicolor flow cytometer (Dako Cytomation). PrP^C^ expression was reported relative to that for untreated N2a cells.

### Liposome protection assay

100 pmol of siRNA was incubated with 0, 10, 100 or 1000 pmol of liposomes at room temperature and 1000 pmol RVG-9r peptide was added ten minutes later. Five microliters of the siRNA-liposome-peptide complexes (LSPCs) were added to 45 µl of freshly isolated mouse serum. Samples were incubated at 37°C and 10 µl aliquots removed at 0, 1, 2 and 4 hours and electrophoresed through a 2% agarose gel stained with ethidium bromide. Bands were visualized and digitally photographed using the LAS 3000 gel documentation system (Fuji).

### LSPC formation

100 pmol of PrP or control siRNA was incubated with 100 pmol of liposomes at room temperature and 1000 pmol of RVG-9r or RVM-9r peptide was added ten minutes later. Liposome size and charge were estimated using a Brookhaven 90Plus particle sizer and a zaetaPlus pontentiometer, respectively (Brookhaven Institute, Holtsville, NY).

### LSPC delivery and PrP^C^ silencing in vitro

1 mL of cell culture medium without antibiotics was added to the LSPCs, which were added to 10^5^ transformed or 10^6^ primary cells five minutes later. Fluorochrome-labeled LSPCs were prepared and used as indicated. Additional control cells received media lacking any of the 3 LSPC components. All cells were incubated at 37°C for 1–4 h. Cells were either harvested and analyzed for peptide binding, siRNA delivery and/or PrP^C^ expression; or supplemented with an additional 1 mL of cell culture media with antibiotics and incubated an additional 16–44 h before analyses. For peptide competition experiments, the indicated molar excess of unlabeled peptide was added to cells suspended in FACS buffer for the indicated times, then analyzed by FACS.

### Prion infection of neuronal cell lines

N2a and NCerP cells were infected with the Rocky Mountain Lab strain of mouse-adapted scrapie prions and E2 CWD prions, respectively, as previously described [37] with slight modifications. Briefly, prion-containing brain homogenates were incubated with 50 µg/mL protease K (PK) for thirty minutes at 37°C, ten minutes at 95°C and then on ice for five minutes. 10^5^ cells were pelleted then resuspended in 100 µl of a 10^−3^ dilution of prions for thirty minutes at room temperature, diluted into 1 mL RPMI medium, 10% FBS and 1X Pen-Strep and incubated at 37°C in each well of a 12-well plate until confluent, then split 1∶3. Confluent cells were split 1∶3 twice more, 1∶10 three times, then analyzed for PrP^RES^ by PK digestion and western blotting (WB) as previously described [38]. Three positive wells from each cell line were expanded and at least ten 5×10^6^-cell aliquots stored in liquid nitrogen.

### LSPC treatment of prion infected cells in vitro

LSPCs were complexed as described and delivered to 10^5^ infected cells passaged ten times. Cells were grown to confluency and split 1∶10 an additional eight times. 90% of cells at each passage were pelleted and assayed for PrP^RES^ by PK digestion and WB. Twenty-five microliters of samples lacking PrP^RES^ and one PrP^RES^+ sample diluted 1000-fold were subjected to six rounds of protein misfolding cyclic amplification (PMCA), PK digestion and WB as previously described [40].

### Analysis of LSPC delivery in vivo

Four nanomoles of Alexa 488-labeled PrP or control siRNA was incubated with 4 nmol of liposomes for twenty minutes at room temperature and 40 nmol of DyLight 488-labeled RVG-9r or RVM-9r peptide added ten minutes later. LSPC volumes were increased to 300 µl with 1X PBS and injected into tail veins of FVB mice. PBS alone was injected into additional control mice. Mice were euthanized 24 hours later, dissected and brains, spleens, livers and kidneys harvested for FACS analysis. An approximately 1 mm coronal brain section through the midbrain was used for FACS analysis, and the remainder was snap-frozen on liquid nitrogen for immunofluorescence microscopy.

### Immunofluorescent staining and microscopy

Where indicated, transformed and primary cells were stained with DyLight 649-labeled Bar224 or POM-2 [71] anti-PrP antibodies as described for FACS analysis. Nuclei were stained with 100 ng/mL DAPI for ten minutes. 10^5^ N2a cells were grown and stained on cover slips and 10^6^ primary cells stained in eppendorf tubes and cytopsun onto glass slides.

For brain sections, 8 µm-thick cryosections were adhered to glass slides, fixed for ten minutes in acetone and air-dried overnight. Slides were incubated in a humidified chamber with 10% FBS and mouse serum for thirty minutes, a 1∶100 dilution of DyLight 649-labeled POM-2 for one hour and 100 ng/mL DAPI for twenty minutes and washed 5X with PBS.

Slides were mounted with coverslips using ProLong Gold antifade fluorescent mounting medium (Invitrogen) and dried at room temperature overnight then at −20°C for 24 h. Cells and sections were visualized and digitally photographed using a BX60 microscope equipped with a DP-71 charge-coupled diode (CCD) camera (Olympus), or an Olympus IX71 microscope using Retiga 2000R CCD camera (Qimaging, Surrey, BC, Canada). Images were acquired using slidebook software (Intelligent Imaging Innovations, Inc., Denver, CO) on a Macintosh G5 dual processor computer (Apple Computer).

### Densitometric and statistical analyses

Densitometric analyses were performed on unsaturated western blots using Quantity One software (Bio-Rad) as previously described [40]. One-way ANOVA with Tukey post-test analysis was performed using GraphPad Prism.
